# The complete plastome sequence of *Platanthera japonica* (Orchidaceae): an endangered medicinal and ornamental plant

**DOI:** 10.1080/23802359.2019.1704643

**Published:** 2020-01-10

**Authors:** Xiao Zhang, Huaizhu Li, Huan Huang, Qiaoli Wang

**Affiliations:** School of Chemistry and Chemical Engineering, Xianyang Normal University, Xianyang City, PR China

**Keywords:** Plastome, *Platanthera japonica*, Illumina sequencing, phylogeny

## Abstract

*Platanthera japonica* (Thunb. ex A. Marray) Lindl belongs to the genus *Platanthera* within family Orchidaceae, is an endangered herbal species in the East Asia area. In this study, the complete plastome sequence (cpDNA) of *P. japonica* was determined by next-generation Illumina sequencing. The cpDNA of this herbal plant is 155,409 bp in size, with a pair of inverted repeat (IR) regions of 26,933 bp that separate a large single-copy (LSC) region of 84,049 bp and a small single-copy (SSC) region of 17,494 bp. The GC content in plastome is 36.9%, and the IR region (43.2%) is higher than that of the LSC and SSC region (34.4% and 29.7%, respectively), which is similar with other Orchidaceae plastomes. The assembled plastome encoded 133 genes, which included 38 tRNA genes, 8 rRNA genes, and 87 protein-coding genes. A total of 24 species were used to construct the phylogenetic relationships among *P. japonica* and other related species within Orchidaceae. The results showed that *P. japonica* is closely related to *Platanthera chlorantha*.

The family Orchidaceae containing six subfamilies about 25,000 species of 725 genera is one of the biggest families about extant angiosperm groups on earth. The Orchidaceae plants are mainly herbaceous taxa all over the world (Dressler 1981, 1993). *Platanthera japonica* (Thunb. ex A. Marray) Lindl (Orchidaceae) is a kind of medicinal and ornamental species and has been listed in ‘China Species Red List’ as an endangered plant (Wang and Xie 2004), mainly distributed in China, Korea, and Japan. However, because of some factors (e.g. the unlimited exploiting, the restrictions of their own reproductive mechanism, and habitat destruction), the habitat area of *P. japonica* is shrinking and makes it an endangered plant (Mendonca and Lins 2007; Ren et al. 2012).

Fresh leaves of *P. japonica* were collected in Qinling Mountain (34°06′N, 107°54′E, Shaanxi, China), and voucher herbarium specimen (No: XNU0120190311) was deposited at Xianyang Normal University. Total DNA was extracted by CTAB method (Doyle 1987) using the next-generation sequencing with Illumina Hiseq 2500 platform by Sangon Biotech Company (Shanghai, China). After filtering out the low-quality sequence, the high-quality sequences were left as the clean reads. They were assembled by MIRA version 4.0.2 (Chevreux et al. 2004) and MITObim version 1.8 (Hahn et al. 2013) with the plastome of closely related species *Habenaria pantlingiana* (KJ524104) as the reference. Finally, a total of 278,819 reads have been assembled, and the average coverage is 270.0×. The programs DOGMA (http://dogma.ccbb.utexas.edu/) (Wyman et al. 2004) and Geneious version 8.0.2 (Kearse et al. 2012) were used to annotate the plastome. The annotated plastome sequence of *P. japonica* has been deposited into GenBank with the accession number MN631092.

The circular complete plastome of *P. japonica* is 155,409 bp in size, with a pair of IR regions of 26,933 bp that separates a LSC region of 84,049 bp and a SSC region of 17,494 bp. The GC content in plastome is 36.9%, and the IR region (43.2%) is higher than that of the LSC and SSC region (34.4% and 29.7%), which is similar with other Orchidaceae plastomes. The assembled plastome encodes 133 genes, including 38 tRNA genes, 8 rRNA genes, and 87 protein-coding genes. Among them, 14 genes (*trnA-UGC*, *trnG-GCC*, *trnI-GAU*, *trnK-UUU*, *trnL-UAA*, *trnV-UAC*, *rpl2*, *rps12*, *rps16*, *rpoC1*, *ndhA*, *ndhB*, *petB*, and *atpF*) contain a single intron, and two genes (*ycf3* and *clpP*) contain two introns.

A total of 24 species were used to construct the phylogenetic tree among the main representatives of Orchidaceae species with *Artemisia argyi* (KM386991) as outgroup (Figure 1). Modeltest version 3.7 (Posada and Crandall 1998) was used to determine the best-fitting model (GTR + G) based on the Akaike information criterion. Maximum-likelihood (ML) analysis was performed using RAxML version 7.2.8 (Stamatakis 2006) with 1000 bootstrap replicates. In the ML tree, the bootstrap values were also high, 20 nodes with 100% bootstrap values. The results indicated that *P. japonicais* is closely related to *P. chlorantha*. The complete plastome of *P. japonicais* can be subsequently used for the systematic study and phylogenetic reconstruction of Orchidaceae.

**Figure 1.**
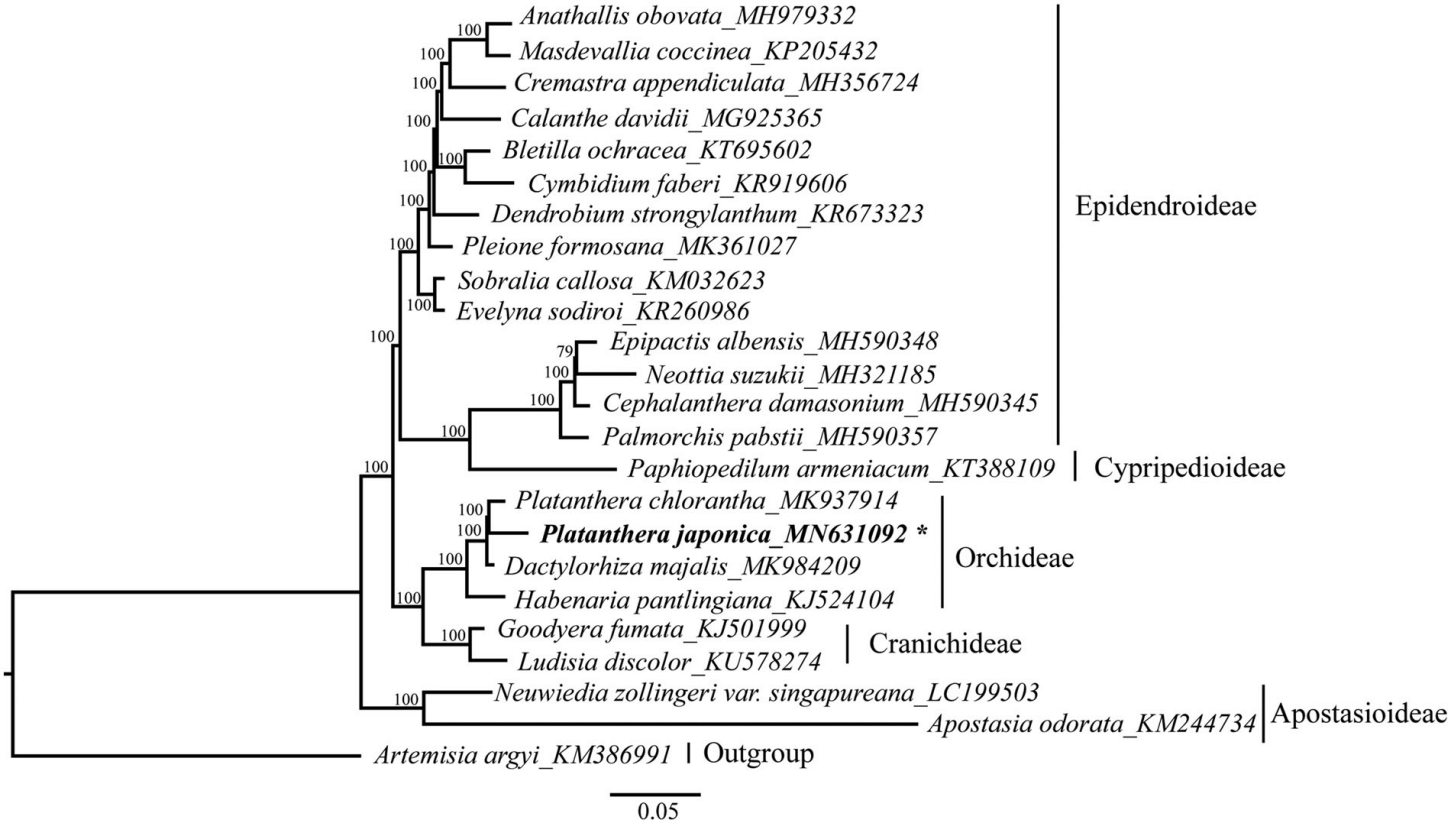
Maximum likelihood (ML) tree of 24 complete plastome sequences: *Anathallis obovata* (MH979332); *Masdevallia coccinea* (KP205432); *Cremastra appendiculata* (MH356724); *Calanthe davidii* (MG925365); *Bletilla ochracea* (KT695602); *Cymbidium faberi* (KR919606); *Dendrobium strongylanthum* (KR673323); *Pleione formosana* (MK361027); *Sobralia callosa* (KM032623); *Evelyna sodiroi* (KR260986); *Epipactis albensis* (MH590348); *Neottia suzukii* (MH321185); *Cephalanthera damasonium* (MH590345); *Palmorchis pabstii* (MH590357); *Paphiopedilum armeniacum* (KT388109); *Platanthera chlorantha* (MK937914); *Platanthera japonica* (MN631092 in this study); *Dactylorhiza majalis* (MK984209); *Habenaria pantlingiana* (KJ524104); *Goodyera fumata* (KJ501999); *Ludisia discolor* (KU578274); *Neuwiedia zollingeri var. singapureana* (LC199503); *Apostasia odorata* (KM244734); and *Artemisia argyi* (KM386991).
